# Is Calcific Tendinitis a Risk Factor for Rotator Cuff Tears? A Retrospective Magnetic Resonance Imaging-Based Case-Control Study

**DOI:** 10.5152/eurasianjmed.2026.251349

**Published:** 2026-01-13

**Authors:** Gökhan Tonkaz, Duygu Erkal, İskender Aksoy, Mehmet Tonkaz, Mehmet Alperen Tezcan, İsmet Miraç Çakır

**Affiliations:** 1 Department of Radiology, Giresun University Faculty of Medicine,Giresun, Türkiye; 2 Department of Emergency Medicine, Giresun University Faculty of Medicine, Giresun, Türkiye; 3 Department of Radiology, Balıkesir University Faculty of Medicine, Balıkesir, Türkiye; 4 Department of Radiology, Samsun University Faculty of Medicine, Samsun, Türkiye

**Keywords:** Calcification, musculoskeletal imaging, rotator cuff tendons, shoulder pain, tear overlap, tendon pathology

## Abstract

**Background:**

The association between calcific tendinitis (CaT) and rotator cuff tears (RCTs) remains controversial, and it is unclear whether CaT represents a predisposing risk factor for tendon tears. This study aimed to investigate the relationship between CaT and RCTs using magnetic resonance imaging (MRI), with a comprehensive evaluation of tear prevalence, tendon involvement, and anatomical overlap patterns between calcifications and tears.

**Methods:**

This retrospective case-control study included 328 patients who underwent shoulder MRI for shoulder pain between 2021 and 2024. The CaT group consisted of 164 patients with CaT detected on MRI and confirmed by radiography or computed tomography, while the control group included 164 consecutive patients without MRI evidence of CaT. Rotator cuff tears were evaluated using established MRI-based classification systems for partial- and full-thickness tears. Tear prevalence, tendon involvement, tear size, degree of retraction, and the anatomical relationship between calcifications and tendon tears were systematically analyzed.

**Results:**

The prevalence of RCTs was significantly lower in the CaT group compared with the control group (21.9% vs. 35.3%, *P* = .01). No significant differences were observed between groups regarding tear type distribution or tendon involvement patterns. Within the CaT group, only 7.3% of calcifications demonstrated direct anatomical overlap with tendon tears in the same segment.

**Conclusion:**

This study demonstrates that CaT does not increase the prevalence of RCTs and that calcifications are predominantly located in anatomical segments independent of tendon tears. The findings indicate that CaT is not a risk factor for RCTs and likely represents an independent biological mineralization process rather than mechanical degeneration.

**Main Points**

- Shoulder pain associated with calcific tendinitis (CaT) can develop in most cases without structural tendon tears and therefore requires careful interpretation in the clinical decision-making process.
- Calcific tendinitis does not increase the prevalence of rotator cuff tears (RCTs); on the contrary, the tear rate was lower in the CaT group than in the control group in the present study.
- No increased risk of concomitant RCTs was observed even in cases with calcifications involving multiple tendons.
- The type of RCTs, the distribution of involved tendons, and retraction patterns are not influenced by the presence of CaT.
- Magnetic resonance imaging plays a critical role in differentiating pseudo-tear appearances associated with CaT from true loss of tendon integrity.

## Introduction

Calcific tendinitis (CaT) is a common shoulder disorder characterized by the deposition of hydroxyapatite within the rotator cuff tendons and is most frequently observed in middle-aged individuals.^1^^,^^2^ The reported prevalence of CaT in the general population ranges from 3% to 8% among asymptomatic individuals, whereas it varies between 33% and 42% in patients presenting with shoulder pain.^3^^-^^5^ Although CaT most commonly involves the supraspinatus tendon, it may also affect the infraspinatus, teres minor, and subscapularis tendons.^6^ Despite typically following a self-limiting course, the disease may, in certain cases, result in severe pain, functional impairment, and sleep disturbance, accounting for a substantial proportion of clinical presentations.^7^

Rotator cuff tears (RCTs) exhibit an age-related increase in prevalence, with chronic degenerative processes, repetitive microtrauma, and alterations in tendon biomechanics contributing to this trend.^8^^,^^9^ Magnetic resonance imaging (MRI) is regarded as the gold standard imaging modality for diagnosis, as it can accurately demonstrate both the low–signal-intensity calcific foci of CaT and the morphological characteristics of RCTs.^10^ However, it has been reported that the inflammatory response and fluid-like signal increases associated with CaT may mimic partial- or full-thickness tears on MRI, potentially leading to reduced specificity.^11^

The relationship between CaT and RCT has long been debated, and the existing literature presents conflicting findings. While some early studies suggested that CaT might be associated with rotator cuff atrophy, hypovascularity, or degeneration and could facilitate the development of RCTs,^12^^,^^13^ radiological and surgical series have demonstrated that the presence of CaT is not associated with an increased incidence of RCTs and does not confer a significant additional risk for RCT development.^14^^-^^16^ Magnetic resonance imaging–based comparative studies have reported that the incidence of RCTs in patients with CaT is not increased compared with those without CaT and has even been found to be lower in some reports.^17^^,^^18^ Nevertheless, the overlap in clinical manifestations between CaT and RCT, particularly during painful phases, complicates the differential diagnosis.^17^

In recent years, detailed MRI-based classification systems have been developed to delineate the anatomical variations, patterns of involvement, and associations with surgical outcomes of RCTs.^10^ These advances underscore the importance of a more refined approach to the characterization of tears occurring in the presence of CaT. Furthermore, in cases where CaT is large or chronic, removal of calcific material has been reported to result in intratendinous defects that may require surgical repair.^19^

In this context, a more detailed and comprehensive understanding of the relationship between CaT and RCTs is clinically important for improving diagnostic accuracy and guiding appropriate treatment strategies. In this study, by systematically combining MRI-based standardized RCT classification systems with a segment-based anatomical overlap analysis, tear prevalence, involved tendon distribution, tear morphology, retraction characteristics, and the anatomical relationship between calcifications and tendon tears were evaluated in detail. This approach is expected to provide a more refined perspective on the debated relationship between CaT and rotator cuff pathology.

## Material and Methods

### Study Design and Population

In this retrospective case-control study, a total of 2487 shoulder MRI examinations from 2152 adult patients who underwent MRI in the clinic for shoulder pain between October 2021 and December 2024 were retrospectively reviewed. All shoulder MRI examinations performed during the study period were screened. Repeated MRI examinations of the same shoulder in the same patient (n = 174) were excluded, and only the initial MRI examination for each shoulder was included in the analysis.

**Figure 1.**
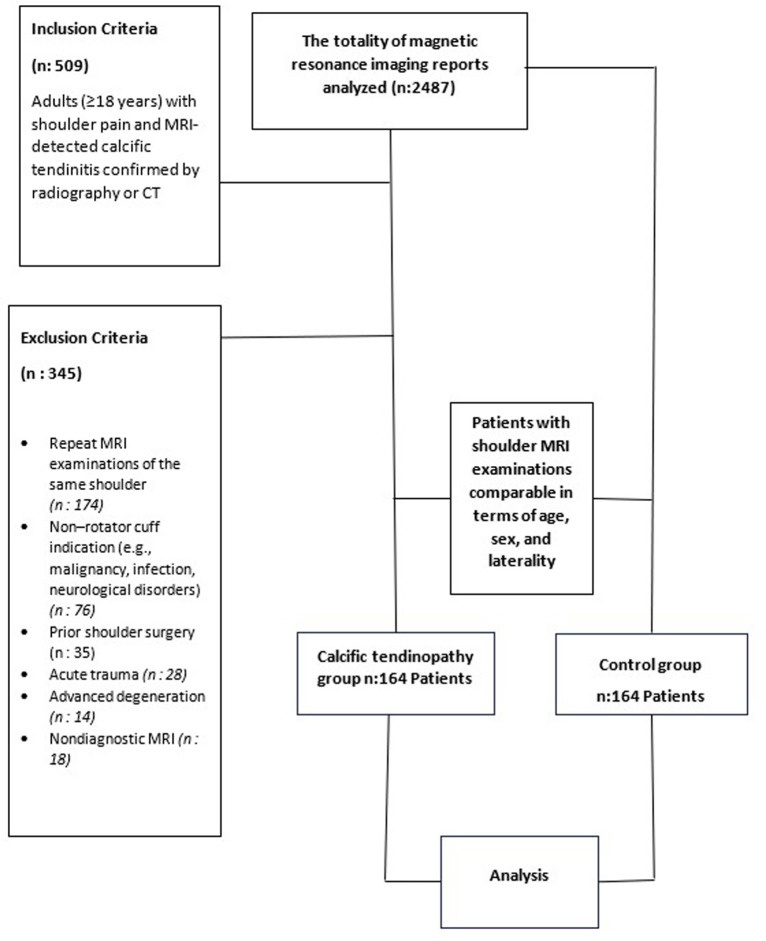
Study flow chart.

Examinations performed for clinical indications other than rotator cuff pathology—such as suspected malignancy (n = 14), infection (n = 8), or neurological complaints including cervical radiculopathy, brachial plexopathy, or peripheral nerve involvement (n = 54)—were excluded. Additional exclusion criteria comprised MRI studies with insufficient diagnostic quality due to severe motion artifacts (n = 18), a history of prior shoulder surgery (n = 35), acute trauma (n = 28), and the presence of advanced degenerative disease that could interfere with rotator cuff evaluation (n = 14). After application of these exclusion criteria, 164 patients with CaT identified on MRI and confirmed by plain radiography or computed tomography (CT) constituted the CaT group. During the same period, 164 consecutive patients who met the inclusion criteria and demonstrated no MRI evidence of CaT were enrolled as the control group (Figure 1).

This study was conducted using a retrospective design without any additional interventions beyond the evaluation of shoulder MRI images and patient records. The research was performed in accordance with the principles of the Declaration of Helsinki and was approved by the Giresun Training and Research Hospital Clinical Research Ethics Committee (approval number: BAEK-227). All data were anonymized, and confidentiality and data security were ensured in compliance with standards recommended in the literature. Owing to the retrospective nature of the study, the ethics committee waived the requirement for informed consent.

### MRI Protocol

All shoulder MRI examinations were performed using standardized protocols. All shoulder MRI examinations were performed using a 1.5-Tesla Siemens Aera scanner (Siemens Healthineers, Germany, 2016). The imaging protocol included fat-suppressed fluid-sensitive sequences as well as T1- and proton density–weighted sequences obtained in the coronal, sagittal, and axial planes.^20^ These sequences allow accurate assessment of the low–signal-intensity features characteristic of CaT and the morphological alterations associated with RCTs.

### RCT Classification

Rotator cuff tears were evaluated using universally accepted classification systems based on anatomical surface involvement, the extent of tendon thickness loss, and morphological features that determine the clinical relevance of the tear. Partial-thickness tears of the supraspinatus and infraspinatus tendons were classified according to their location as articular-sided, bursal-sided, or intratendinous, and were graded based on the proportion of tendon thickness involved as Grade 1 (<25%), Grade 2 (25%-50%), and Grade 3 (>50%).^21^ This approach allows standardized characterization of partial-thickness tears in terms of both surface involvement and depth, in accordance with classification systems recommended in the literature.

Full-thickness tears involving the supraspinatus and infraspinatus tendons were evaluated together, as they share similar biomechanical characteristics due to complete loss of tendon thickness, and were categorized according to the Cofield classification based on tear size as C1 (<1 cm), C2 (1-3 cm), C3 (3-5 cm), and C4 (>5 cm).^22^

For full-thickness tears of the supraspinatus and infraspinatus tendons, the degree of retraction was assessed using the Patte classification because of its prognostic relevance. Tendon retraction was measured on coronal fat-suppressed T2-weighted images. According to the Patte classification, Grade 1 refers to minimal tendon retraction, with the tear margin located near the lateral edge of the humeral head; Grade 2 indicates that the tendon edge has retracted medial to the highest point of the humeral head; and Grade 3 describes retraction of the tendon fibers beyond the humeral head to the level of the superior glenoid margin or more proximally.^22^

Subscapularis tendon tears were evaluated using the Lafosse classification, which describes an anatomical progression ranging from involvement of the superior tendon fibers to complete tendon disruption, and were graded as Types 1 through 5. Within this system, only Type 1 lesions were considered partial-thickness tears, whereas Type 2 lesions—characterized by complete detachment of the upper third of the tendon—and the more advanced structural disruptions represented by Types 3, 4, and 5 were classified as full-thickness tear.^22^

### Image Evaluation

The images were independently reviewed by 2 radiologists with experience in musculoskeletal imaging. In cases of disagreement between the observers, the opinion of a third radiologist was sought, and the final decision was reached by consensus. Interobserver agreement assessment was performed on a tendon-based approach for the detection of the presence of RCTs.

The diagnosis of CaT was based on the presence of globular or linear calcific deposits demonstrating markedly low signal intensity across all sequences, with confirmation by plain radiography or CT findings.

The diagnosis of RCT was established on the basis of morphological features, including disruption of tendon continuity, discontinuity of tendon fibers, focal or diffuse thinning of tendon thickness, fluid signal intensity along the tear margin, and cortical surface irregularity. Given that inflammatory signal changes surrounding CaT may mimic the appearance of partial-thickness tears, demonstration of true disruption of tendon integrity was considered the essential criterion for the diagnosis of RCT.

### Statistical Analysis

Statistical analyses were performed using SPSS Statistics for Windows, Version 26.0 (IBM Corp., Armonk, NY). Continuous variables are summarized as mean ± standard deviation, and differences between the 2 groups were assessed using the independent samples *t*-test. Categorical variables are expressed as counts and percentages, and the chi-square test was applied to evaluate overall differences in distributions between groups. When more than 20% of the expected cell frequencies were <5 or when cell counts were small—particularly in comparisons of subclassified RCT types—Fisher’s exact test was used. In all statistical analyses, a *P* value < .05 was considered statistically significant. Interobserver agreement for the presence or absence of RCTs in the supraspinatus, infraspinatus, and subscapularis tendons was calculated using Cohen’s kappa coefficient. Kappa values were interpreted according to the Landis and Koch classification.

## Results

A total of 328 patients were included in the study, comprising 164 patients diagnosed with CaT and 164 control individuals without evidence of CaT. No statistically significant differences were observed between the CaT and control groups with respect to age or sex distribution. Likewise, no significant differences were found between the 2 groups in terms of comorbidity profiles, including hypertension, diabetes mellitus, hypothyroidism, connective tissue diseases, and inflammatory/rheumatoid arthritis (all *P* > .05) (Table 1).

When the side of shoulder involvement was evaluated, right shoulder involvement was identified in 58.2% of all cases (n = 191), with no statistically significant difference between the CaT and control groups regarding the affected side (*P* = .372) (Table 1).

In the CaT group, the mean calcification diameter was 11.5 ± 5.7 mm. Analysis of the anatomical distribution of calcifications revealed that the supraspinatus tendon was most frequently involved (42.1%; n = 69), followed by the infraspinatus (26.2%; n = 43) and subscapularis (18.9%; n = 31) tendons. No calcifications were detected in the teres minor tendon. Additionally, multiple calcifications involving both the supraspinatus and infraspinatus tendons were observed in 12.8% of cases (n = 21) (Table 1).

Interobserver agreement analysis was conducted to determine the presence or absence of RCTs in the supraspinatus, infraspinatus, and subscapularis tendons. Agreement between the independent assessments of 2 experienced radiologists was calculated on a tendon-based level using Cohen’s kappa coefficient. Accordingly, interobserver agreement was *κ* = 0.81 for the supraspinatus tendon, *κ* = 0.74 for the infraspinatus tendon, and *κ* = 0.86 for the subscapularis tendon. These findings indicate good-to-very good interobserver agreement in the assessment of RCT presence across all rotator cuff tendons.

The prevalence of RCTs was identified in 36 patients (21.9%) in the CaT group and in 58 patients (35.3%) in the control group, with the difference between groups being statistically significant (*P* = .010). In subgroup analyses according to tear type, the rate of partial-thickness tears was 12.2% in the CaT group and 20.7% in the control group; however, this difference did not reach statistical significance (*P* = .070). Similarly, full-thickness tear rates were 9.8% and 14.6% in the CaT and control groups, respectively, with no significant difference between groups (*P* = .230) (Table 1).

Among the 94 patients with RCTs, tears were most frequently located in the supraspinatus tendon (64.9%; n = 61), followed by the infraspinatus (19.1%; n = 18) and subscapularis (16.0%; n = 15) tendons. In tendon-based comparisons, supraspinatus tears were observed in 14.6% of patients in the CaT group and 22.6% in the control group, with no statistically significant difference between groups (*P* = .090). Likewise, no significant intergroup differences were found for infraspinatus (CaT: 4.3%; control: 6.7%; *P* = .460) or subscapularis tendon tears (CaT: 3.0%; control: 6.0%; *P* = .280).

The overall prevalence of supraspinatus tendon tears in the study population was 18.6% (n = 61). The stage distribution (Grade 1-3) and surface location (articular-sided, bursal-sided, and intratendinous) of partial-thickness supraspinatus tears were comparable between the CaT and control groups, with no statistically significant differences observed for any stage (all *P* > .05). Full-thickness supraspinatus tendon tears were evaluated according to tear size using the Cofield classification and according to retraction using the Patte classification; no significant differences were identified between the CaT and control groups with respect to C1-C4 tear categories or their corresponding retraction patterns (*P* > .05) (Table 2).

The prevalence of infraspinatus tendon tears in the study population was 5.5% (n = 18). The stage distribution and surface location of partial-thickness infraspinatus tears were similar between the CaT and control groups, and no statistically significant differences were observed between groups in the distribution of full-thickness tears according to the Cofield and Patte classifications (all *P* > .05) (Table 2).

Subscapularis tendon tears were identified in 4.6% of the study population (n = 15). According to the Lafosse classification, partial-thickness tears were limited to Type 1 lesions, with no significant difference between the CaT and control groups for this subtype. Full-thickness subscapularis tendon tears were detected in a limited number of cases within the Type 2-4 range, and no Type 5 tears were observed. No statistically significant differences were found between groups in the distribution of Lafosse tear types overall (*P* > .05) (Table 2).

**Figure 2.**
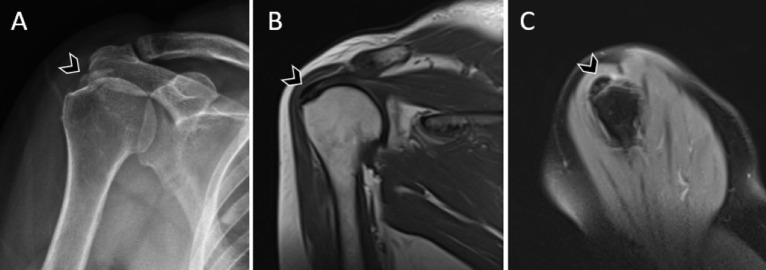
Calcific tendinitis of the supraspinatus tendon without an associated tear. Right shoulder images of a 50-year-old female patient. (A) Plain radiograph of the right shoulder demonstrates an approximately 15 mm calcific radiopaque focus at the supraspinatus tendon insertion (arrowhead). (B) Coronal T1-weighted magnetic resonance imaging shows a corresponding low-signal-intensity calcific focus at the same location (arrowhead). (C) Sagittal proton density–weighted image clearly demonstrates the calcific deposit (arrowhead), with no definite evidence of a supraspinatus tendon tear (arrowhead).

**Figure 3.**
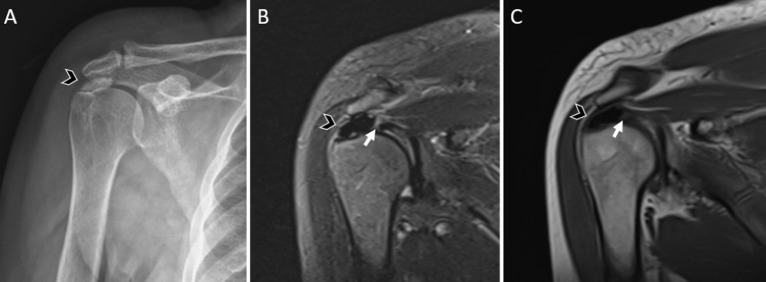
Calcific tendinitis accompanied by a tear in the same tendon and at the same anatomical location. Right shoulder images of a 60-year-old male patient.(A)Plain radiograph of the right shoulder demonstrates an approximately 17 mm calcific radiopaque focus at the supraspinatus tendon insertion (arrowhead).(B)Sagittal proton density–weighted and(C)coronal T1-weighted magnetic resonance imaging images show a low-signal-intensity calcific focus at the same location (arrowhead), together with a full-thickness tear of the supraspinatus tendon at the same anatomical level (arrow).

**Figure 4.**
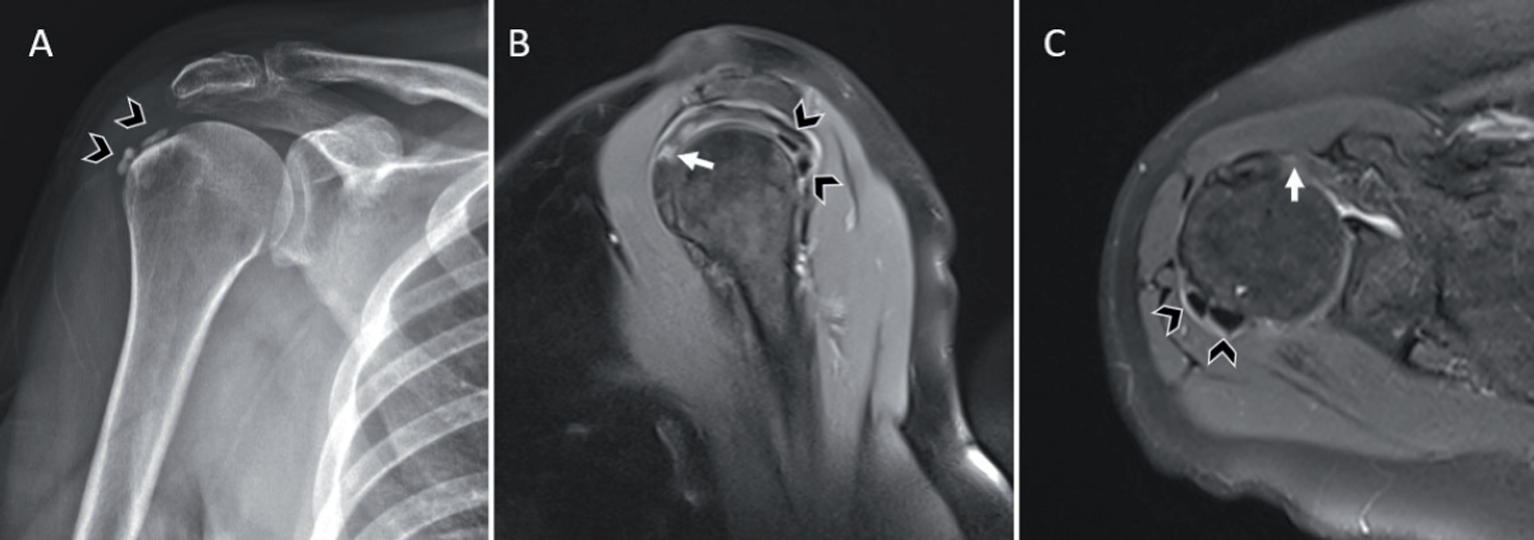
Calcific tendinitis associated with a tear in a different tendon. Right shoulder images of a 51-year-old female patient.(A)Plain radiograph of the right shoulder demonstrates 2 calcific radiopaque foci adjacent to the humerus, the largest measuring approximately 10 mm in diameter (arrowheads).(B)Sagittal proton density (PD) and(C)axial PD-weighted magnetic resonance imaging images show 2 calcific deposits within the infraspinatus tendon, the largest measuring approximately 10 mm (arrowheads). In the same images, an articular-sided partial-thickness tear of the subscapularis tendon is identified (arrow).

**Figure 5.**
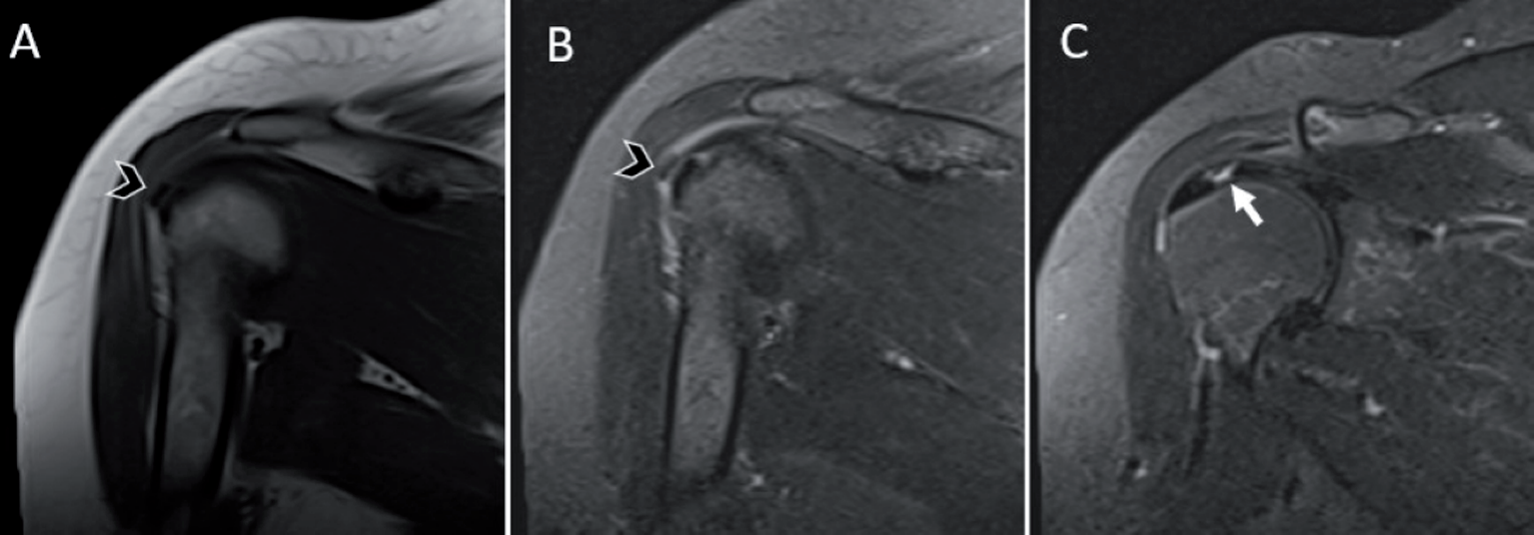
Calcific tendinitis associated with a tear at a different location within the same tendon. Right shoulder images of a 60-year-old female patient.(A)Coronal T1-weighted and (B)coronal proton density–weighted magnetic resonance imaging (MRI) images demonstrate an approximately 7 mm calcific focus at the humeral insertion of the supraspinatus tendon (arrowhead).(C)A different coronal T1-weighted MRI image shows an approximately 5-mm articular-sided partial-thickness tear of the supraspinatus tendon (arrow).

When the relationship between calcification location and RCTs was evaluated in the CaT group, the majority of calcifications were not associated with concomitant tendon tears (Figure 2). No tear was observed in 65.2% of calcifications located in the supraspinatus tendon, 83.7% of those in the infraspinatus tendon, and 83.9% of those in the subscapularis tendon. In cases with multiple calcifications involving both the supraspinatus and infraspinatus tendons, no tears were identified in any patient. Among cases in which tears were present, the rate of complete anatomical overlap between calcification and tear within the same tendon and the same anatomical location was low (Figure 3); tears were more commonly located in a different tendon (Figure 4) or in a different segment of the same tendon (Figure 5). Overall, 78.0% of calcifications in the CaT group were not associated with any tear, and only 7.3% demonstrated exact anatomical overlap between calcification and tendon tear (Table 3).

## Discussion

This study represents one of the more comprehensive evaluations of the association between CaT and RCTs, addressing both tear prevalence and patterns of anatomical overlap. The findings demonstrated that CaT was most frequently localized to the supraspinatus tendon. This distribution is consistent with previous studies reporting that the supraspinatus tendon is particularly susceptible to CaT development due to the presence of an avascular critical zone and chronic mechanical loading within the subacromial space.^17^^,^^22^ However, the observation that calcific deposits were predominantly not located within the same anatomical segments as mechanically damaged or torn tendon regions suggests that CaT formation is not directly related to focal mechanical stress or degenerative tear sites. Indeed, perspectives in the literature proposing that CaT preferentially develops in relatively stable tendon segments—where cellular metabolic and biochemical processes predominate rather than overt degenerative change—are in line with the anatomical overlap patterns observed in the study.^23^^,^^24^

The significantly lower rate of RCTs observed in the CaT group compared with the control group is consistent with the contemporary biological model, which posits that CaT may not be a predisposing condition for tendon tearing. High-resolution imaging studies have indicated that CaT is associated with fibroblast differentiation, cellular stress, and transformation of the calcified matrix, rather than with direct degenerative fiber loss.^18^^,^^25^ Although some clinical series have proposed that CaT may increase the risk of tearing by augmenting mechanical loading,^26^ prospective and cross-sectional studies have reported no consistent causal relationship between CaT and tendon tears.^11^^,^^27^ In this context, the findings of the present study are aligned with the concept that CaT likely represents an independent biological mineralization process rather than a manifestation of mechanical degeneration.

The very low rates of calcification–tear colocalization within the same anatomical segment, as illustrated in Figures 4 and 5 where calcifications and tears are located in distinct regions, suggest a limited local association between CaT and tendon integrity. In the supraspinatus tendon, the finding that calcifications overlapped with tears in only 11.6% of cases indicates that CaT is not directly associated with regions of mechanical stress but rather tends to arise in more stable tendon segments.^22^ Similarly, the low rates of overlap between CaT and tears in the infraspinatus and subscapularis tendons suggest that CaT reflects a localized biochemical response rather than a multifocal degenerative process.^28^

The similarity in retraction patterns of full-thickness tears between the CaT and control groups in the study suggests that CaT may not substantially alter tendon biomechanics in the context of a full-thickness tear. Given that the degree of retraction according to the Patte classification is recognized as one of the strongest predictors of tear prognosis, the absence of any modifying effect of calcification on this behavior is consistent with previous studies reporting that CaT does not significantly influence tendon elasticity or tensile capacity.^18^^,^^24^^,^^26^ This finding is consistent with the notion that CaT may not be associated with mechanical compromise of tendon integrity.

The lower involvement rates of the subscapularis tendon in both CaT and RCTs may be explained by load distribution within shoulder biomechanics and the distinct functional roles of the rotator cuff tendons. Although the infraspinatus tendon is affected less frequently than the supraspinatus, it demonstrates a higher prevalence of RCTs compared with the subscapularis. The supraspinatus tendon has been extensively reported in the literature as being the most susceptible to pathology, owing to its sensitivity to axial loading and its stabilizing role over the humeral head.^22^^,^^27^ Notably, the absence of tears even in cases with multi-tendon calcifications (Figure 2 for an example of a sizable calcification without tear) suggests that the multifocal presence of CaT may not be associated with a meaningful risk to tendon integrity and is consistent with the contemporary view that CaT may not be directly involved in tear pathogenesis.^28^

The pathophysiology of CaT is recognized as a process comprising distinct biological phases, as described by Uhthoff and Loehr, in which clinical symptoms become particularly prominent during the resorptive phase, whereas the pre-calcific and resting phases may often follow an asymptomatic course. However, the existing literature does not provide consistent evidence demonstrating that specific phases of CaT are directly associated with the development of RCT or that CaT leads to asymptomatic tendon tears.^29^ The design of the study, which focused on a symptomatic patient population, limited the ability to comprehensively evaluate the relationship between all phases of CaT (particularly asymptomatic forms) and RCT. Consequently, the present findings primarily reflect the association between CaT and RCT within the context of patients presenting with clinically significant shoulder pain.

This study also demonstrates that pain associated with CaT develops independently of tendon tearing in most cases and that the presence of pain alone is not indicative of a tendon tear. The literature consistently reports that CaT can cause severe pain during symptomatic phases without compromising tendon integrity.^17^^,^^22^^,^^25^ In this context, the findings reaffirm that clinical symptoms should not be conflated with the presence of anatomical tendon tears.

### Limitations

This study has several limitations. Owing to its retrospective design, clinical parameters such as symptom duration, pain severity, and functional scores could not be obtained in a standardized manner. Although the presence of calcification was confirmed by plain radiography or CT, advanced quantitative analyses of calcification density, maturation stage, and morphological characteristics could not be performed. The absence of a separate phase-based classification of the biological stages of CaT limited the evaluation of potential associations between RCT and specific CaT phases, particularly the asymptomatic stages. The absence of arthrographic MRI protocols may have limited more detailed characterization of partial-thickness tendon tears and intra-articular surface pathology. In addition, because radiological findings were not validated by arthroscopic correlation, the concordance between imaging findings and specific anatomical lesion subtypes could not be directly assessed. The single-center design of the study and minor technical variations in MRI sequences also partially limit the generalizability of the results. Larger, prospective, and arthroscopy-based studies are warranted.

### Clinical Implications

This study provides important insights into the clinical management of CaT. Demonstrating that CaT can cause severe pain during symptomatic periods without compromising tendon integrity may reduce the likelihood of clinicians attributing pain to structural tendon disruption and, consequently, help prevent unnecessary surgical interventions. Furthermore, the lack of a significant effect of CaT on tendon biomechanics indicates that CaT should not be considered a standalone risk factor for tendon tearing in treatment planning. For both radiologists and orthopedic surgeons, recognizing CaT as an independent pathology that is not directly involved in the pathogenesis of tendon tears may help avoid false-positive surgical decision-making.

### Future Directions

Future studies would benefit from evaluating the biological subtypes of CaT, the structural properties of calcific material, and its interaction dynamics with tendon tissue using advanced imaging and histopathological methods. Prospective investigations may further elucidate the temporal evolution of CaT, the impact of calcification stiffness on tendon biomechanics, and the relationship between symptoms and tendon tears. In addition, artificial intelligence–based segmentation and tissue characterization approaches may offer novel insights into the pathophysiology of CaT.

## Conclusion

This study investigated the relationship between CaT and RCTs through a detailed analysis of tear prevalence, tendon involvement, and patterns of anatomical overlap between calcifications and tears. The findings demonstrate that CaT does not increase the frequency of RCTs and that calcifications are predominantly located in regions independent of tendon tears. The similarity in retraction grades of full-thickness tears between the CaT and control groups further supports the absence of a meaningful effect of CaT on tendon biomechanics or structural integrity. Collectively, these results indicate that CaT reflects an independent biological mineralization process rather than mechanical degeneration.

The imaging-based findings show that CaT does not compromise tendon integrity and also suggest that symptoms in patients presenting with shoulder pain are not invariably attributable to RCTs. Awareness that CaT alone can cause substantial pain facilitates accurate identification of the pain source and helps prevent diagnostic errors and unnecessary surgical referrals that may arise from incorrectly attributing symptoms to RCTs.

In conclusion, this study strengthens the evidence that CaT is not directly involved in the pathogenesis of RCTs and provides an important reference for the accurate interpretation of CaT, particularly in MRI-based assessments.

## Figures and Tables

**Table 1.** Comparison of Demographics and Incidence of Rotator Cuff Tear Between the 2 Groups

	**Total (n = 328)**	**CaT Group (n = 164)**	**Control Group (n = 164)**	***P***
Age	54.8 ± 8.5	54.8 ± 8.4	54.9 ± 8.7	.916^I^
Gender n (%)				
Female	192 (58.5)	95 (57.9)	97 (59.1)	.911^C^
Male	136 (41.5)	69 (42.1)	67 (40.9)	
Comorbidity n (%)				
Hypertension	29 (8.8)	17 (10.4)	12 (7.3)	.437^C^
Diabetes	26 (7.9)	15 (9.1)	11 (6.7)	.540^C^
Hypothyroidism	12 (3.7)	7 (4.3)	5 (3.0)	.769^C^
Connective tissue disease	7 (2.1)	4 (2.4)	3 (1.8)	1.000^F^
Inflammatory/Rheumatoid Arthritis	5 (1.5)	3 (1.8)	2 (1.2)	1.000^F^
Diseased side of shoulder joint n (%)				
Right	191 (58.2)	91 (55.5)	100 (61)	.372^C^
Left	137 (41.8)	73 (44.5)	64 (39)	
Calcification diameter (mm)	—	11.5 ± 5.7	—	
Calcific lesion location n (%)			—	
Supraspinatus tendon	—	69 (42.1)	—	
Infraspinatus tendon	—	43 (26.2)	—	
Subscapularis tendon	—	31 (18.9)	—	
Supraspinatus and infraspinatus tendons	—	21 (12.8)	—	
Rotator cuff tear n (%)				
Yes	94 (28.7)	36 (21.9)	58 (35.3)	**.010** **^C^**
No	234 (71.3)	128 (78.1)	106 (64.7)	
Size of rotator cuff tear n (%)	94 (28.7)	36 (21.9)	58 (35.3)	
Partial tear	54 (16.5)	20 (12.2)	34 (20.7)	.070^C^
Fullthickness tear	40 (12.2)	16 (9.8)	24 (14.6)	.230^C^
Site of rotator cuff tear n (%)	94 (28.7)	36 (21.9)	58 (35.3)	
Supraspinatus tendon	61 (18.6)	24 (14.6)	37 (22.6)	.090^C^
Infraspinatus tendon	18 (5.5)	7 (4.3)	11 (6.7)	.460^C^
Subscapular tendon	15 (4.6)	5 (3.0)	10 (6.0)	.280^C^

Categorical variables are presented as n (%), and continuous variables are presented as mean ± standard deviation. n denotes the number of patients, and mm denotes millimeters. *P* values are provided for the appropriate comparisons.

^I^Calculate the *P* value using the independent t-test.

^C^Calculate the *P* value using the chi-square test.

^F^Calculate the *P* values using Fisher’s exact test.

**Table 2.** Distribution of the Types and Grades of Tears Identified in the Supraspinatus, Infraspinatus, and Subscapularis Tendons in the Calcific Tendinitis Group and the Control Group

	**Total (n = 328)**			**CaT Group (n = 164)**			**Control Group (n = 164)**			***P***
Supraspinatus tendon, n (%)	61 (18.6)			24 (14.6)			37 (22.6)			.090^C^
**Partial tear, n**	**A**		**B**		**I**	**A**		**B**		**I**	**A**	**B**		**I**	
Grade 1*	11		5		4	4		2		2	7	3		2	.333^C^
Grade 2*	7		2		2	2		1		1	5	1		1	.363^C^
Grade 3*	4		2		1	2		1		0	2	1		1	1.000^F^
**Full thickness tear, n**	**R I**		**R II**		**R III**	**R I**		**R II**		**R III**	**R I**	**R II**		**R III**	
C1**	10		0		0	4		0		0	6	0		0	1.000^F^
C2**	3		1		0	1		1		0	2	0		0	1.000^F^
C3**	0		3		0	0		1		0	0	2		0	1.000^F^
C4**	0		0		6	0		0		2	0	0		4	1.000^F^
Infraspinatus tendon, n (%)	18 (5.5)			7 (4.3)			11 (6.7)			.46^C^
**Partial tear, n**	**A**	**B**		**I**		**A**	**B**		**I**		**A**		**B**	**I**		1.000^F^
Grade 1*	3	1		1		1	1		0		2		0	1		1.000^F^
Grade 2*	2	0		0		1	0		0		1		0	0		1.000^F^
Grade 3*	1	0		0		0	0		0		1		0	0		1.000^F^
**Full thickness tear, n**	**R I**	**R II**		**R III**		**R I**	**R II**		**R III**		**R I**		**R II**	**R III**		
C1**	3	0		0		1	0		0		2		0	0		1.000^F^
C2**	1	1		0		0	1		0		1		0	0		1.000^F^
C3**	0	1		0		0	0		0		0		1	0		1.000^F^
C4**	0	0		4		0	0		2		0		0	2		1.000^F^
Subscapular tendon, n (%)	15 (4.6)			5 (3.0)			10 (6.0)			.281^C^
**Partial tear, n**										
**Lafosse Tip 1**	8			2			6			.273^F^
**Full thickness tear, n**										
**Lafosse Tip 2**	2			1			1			1.000^F^
**Lafosse Tip 3**	2			1			1			1.000^F^
**Lafosse Tip 4**	3			1			2			1.000^F^
**Lafosse Tip 5**	0			0			0			—

Categorical variables are presented as n and n (%). n denotes the number of patients. For subgroups with limited sample sizes, percentage values were not calculated to avoid potentially misleading interpretations. *P* values are provided for the appropriate comparisons. A, articular-sided; B, bursal-sided; I, intrasubstance; R, retraction – Patte classification..

^C^Chi-square (Pearson) test.

^F^Fisher Exact test.

*Ellman (based on tear depth).

**Cofield Classification (based on tear size).

**Table 3.** Distribution of Calcific Lesions Across Rotator Cuff Tendons and Their Anatomical Relationship with Coexisting Rotator Cuff Tears in the CaT Group

**Calcific Lesion Location **	**Tear Not Present, ** **n (%)**	**Tear Presence, ** **n (%)**	**Tear in a Different Tendon, ** **n (%)**	**Tear in a Different Location Within the Same Tendon, ** **n (%)**	**Tear in the Same Tendon and in the Same Position, ** **n (%)**
Supraspinatus tendon (n = 69)	45 (65.2)	24 (34.8)	10 (14.5)	6 (8.7)	8 (11.6)
Infraspinatus tendon (n = 43)	36 (83.7)	7 (16.3)	4 (9.3)	1 (2.3)	2 (4.7)
Subscapularis tendon (n = 31)	26 (83.9)	5 (16.1)	2 (6.5)	1 (3.2)	2 (6.5)
Supraspinatus–infraspinatus tendons (n = 21)	21 (100)	0 (0)	0 (0)	0 (0)	0 (0)
Total (n = 164)	128 (78.0)	36 (22.0)	16 (9.8)	8 (4.9)	12 (7.3)

Categorical variables are presented as n and n (%). n denotes the number of patients.

## Data Availability

The data that support the findings of this study are available on request from the corresponding author.
